# Identification of Electroencephalogram Signals in Alzheimer's Disease by Multifractal and Multiscale Entropy Analysis

**DOI:** 10.3389/fnins.2021.667614

**Published:** 2021-06-28

**Authors:** Momo Ando, Sou Nobukawa, Mitsuru Kikuchi, Tetsuya Takahashi

**Affiliations:** ^1^Graduate School of Information and Computer Science, Chiba Institute of Technology, Narashino, Japan; ^2^Department of Computer Science, Chiba Institute of Technology, Narashino, Japan; ^3^Department of Psychiatry and Behavioral Science, Kanazawa University, Ishikawa, Japan; ^4^Research Center for Child Mental Development, Kanazawa University, Ishikawa, Japan; ^5^Department of Neuropsychiatry, University of Fukui, Fukui, Japan; ^6^Uozu Shinkei Sanatorium, Uozu, Japan

**Keywords:** EEG signal, Alzheimer's disease, multifractal, multiscale entropy, early diagnosis

## Abstract

Alzheimer's disease (AD) is the most common form of dementia and is a progressive neurodegenerative disease that primarily develops in old age. In recent years, it has been reported that early diagnosis of AD and early intervention significantly delays disease progression. Hence, early diagnosis and intervention are emphasized. As a diagnostic index for AD patients, evaluating the complexity of the dependence of the electroencephalography (EEG) signal on the temporal scale of Alzheimer's disease (AD) patients is effective. Multiscale entropy analysis and multifractal analysis have been performed individually, and their usefulness as diagnostic indicators has been confirmed, but the complemental relationship between these analyses, which may enhance diagnostic accuracy, has not been investigated. We hypothesize that combining multiscale entropy and fractal analyses may add another dimension to understanding the alteration of EEG dynamics in AD. In this study, we performed both multiscale entropy and multifractal analyses on EEGs from AD patients and healthy subjects. We found that the classification accuracy was improved using both techniques. These findings suggest that the use of multiscale entropy analysis and multifractal analysis may lead to the development of AD diagnostic tools.

## 1. Introduction

Alzheimer's disease (AD) is the most common form of dementia and is a progressive neurodegenerative disease that primarily develops in old age (Liu et al., 2014). The World Health Organization estimates that the global prevalence of AD will increase to 0.6% in 2030 and 1.2% by 2046 (Brookmeyer et al., 2007). Although there is no effective treatment for AD, in recent years, it has been reported that early diagnosis of AD and early intervention significantly delay the progression of the disease. Hence, it would be ideal to diagnose AD early in its clinical course (Liu et al., 2014).

In AD, there are three significant anatomical changes: progressive neuronal death, neurofibrillary tangles, and senile plaques in extensive brain areas (Sims et al., 2017; Yamaguchi-Kabata et al., 2018). Positron emission tomography (PET) and magnetic resonance imaging (MRI) are often used to diagnose AD and detect neurotransmitter activity disorders, amyloid beta plaque deposition, and brain atrophy (Ewers et al., 2011; McKhann et al., 2011; Sperling et al., 2011). As methods focused on functional neural activity, studies based on the temporal behavior of neural activity were conducted using electroencephalography (EEG), magnetoencephalography (MEG), and functional magnetic resonance imaging (fMRI) (Greicius et al., 2004; Jeong, 2004; Stam, 2005; Dickerson and Sperling, 2008; Takahashi, 2013; Yang and Tsai, 2013; Wang et al., 2017; Nobukawa et al., 2020).

Among all these evaluations, EEG is cost-effective, widely available, and non-invasive, making it ideal for clinical applications (Vecchio et al., 2013; Kulkarni and Bairagi, 2018). AD's pathological progression alters EEG behavior, such as slow waves, low synchronization of neural activity among brain regions, and low temporal complexity. Complexity analysis is a good approach to detect cortical disconnection in AD because this state impairs mutual neural interaction among widespread brain regions. Studies assessing EEG signals' complexity in patients with AD focused on deterministic chaos and fractal dimensions, such as the correlation dimension and Lyapunov exponent (Kantz and Schreiber, 2004). These studies reported a reduction in the complexity of neural activity in AD patients (Woyshville and Calabrese, 1994; Besthorn et al., 1995; Jelles et al., 1999; Jeong, 2004; Smits et al., 2016; Al-Nuaimi et al., 2017). Moreover, EEG dynamics at each temporal scale and frequency band, such as theta, beta, and gamma bands, are associated with different memory function components, cognitive and perceptual function (Klimesch et al., 2007). Hence, as a diagnostic index for AD patients with various brain function defects, the evaluation of the complexity with temporal scale dependence in EEG signal is effective (Mizuno et al., 2010; Nobukawa et al., 2019, 2020).

Multiscale entropy (MSE) analysis and multifractal (MF) analysis are known as typical temporal scale complexity dependency analyses (Takahashi, 2013; Yang and Tsai, 2013). In addition to EEG's temporal dependency in AD, MSE analysis also showed lower complexity on a small temporal scale in the frontal region in AD; in comparison, higher complexity was observed across this brain region in AD on a larger temporal scale (Mizuno et al., 2010; Ni et al., 2016). Zorick *et al*. reported that a statistical model based on MF analysis could detect clinical stages of severity and degree of progress from cognitive impairment to AD (Zorick et al., 2020). As described above, MSE and MF analyses have a high ability to detect the complexity in EEG signals of AD. As such, these indices might become biomarkers for AD to evaluate the alteration of EEG complexity (Mizuno et al., 2010; Ni et al., 2016; Nobukawa et al., 2020).

Recent studies have focused on the enhancement of classification accuracy combining several feature values, including complexity indexes in EEG of AD patients (Wang et al., 2015; Gómez et al., 2017; Ieracitano et al., 2020; Nobukawa et al., 2020). Particularly, Wang et al. (2015) and Gómez et al. (2017) showed that combinations of spectrum and bispectrum entropy measures enhance the accuracy of EEG signals classification in AD. Therefore, these combinations of complexity measures are a new avenue for the diagnosis of AD EEG signals. Furthermore, Cukic *et al*. showed that multiscale analysis (MSE) and fractal dimension provide complementary information on brain activity in healthy subjects (Cukic et al., 2018). This complementary relationship may enhance the accuracy of AD identification. In this context, we hypothesize that the combination of MSE and fractal analysis may contribute to a better understanding of EEG dynamics' alteration in AD. In this study, we performed MSE analysis and multifractal analysis on the EEGs of patients with AD and healthy controls (HC).

## 2. Materials and Methods

### 2.1. Subject

The subjects of this study were 16 patients with AD and 18 sex-matched and aged-matched healthy old individuals (see Table 1) (Mizuno et al., 2010; Nobukawa et al., 2019, 2020). The sample size of AD and HC groups was determined based on previous works on complexity analysis (Abásolo et al., 2008; Mizuno et al., 2010; Nobukawa et al., 2019, 2020). For this study, we defined healthy old individuals as nonsmokers and not on medication. Subjects with medical or neurological conditions, including epilepsy or head trauma in the past, and subjects with a history of alcohol or drug dependence were excluded. We recruited patients with AD or probable AD who met the NINCDS-ADRDA criteria and in a state before the onset of primary dementia based on DSM-IV criteria. Recruited patients with AD were not receiving medications that act on the central nervous system.

**Table 1:** Physical characteristics of healthy control (HC) and subjects with Alzheimer's disease (AD).

	**HC participants**	**AD participants**	**p-values**
Male/female	7/11	5/11	0.72
Age(year)	59.3 (5.3, 55–66)	57.5 (4.7, 43–64)	0.31
MMSE score	NA	15.5 (4.7, 10–26)	NA

Each patient was evaluated using the Function Assessment Stage (FAST) and Mini-Mental State Examination (MMSE). Three patients had mild dementia (FAST 3); seven moderate dementia (FAST 4); and six severe dementia (FAST 5). The MMSE score ranged from 10 to 26, with an average of 15.56. Table 1 shows subjects' characteristics. All subjects provided informed consent prior to the start of the study. The research protocol was approved by the Ethics Committee of Kanazawa University. All procedures in this study were conducted in accordance with the Declaration of Helsinki.

### 2.2. EEG Recordings

As reported in previous studies, methods have been established to record and preprocess EEG data (Mizuno et al., 2010). When recording the EEG, the participants were seated in an electrically shielded and soundproof recording room, and the room lighting was controlled. For the EEG measurement, 16 electrodes (Fp1, Fp2, F3, F4, C3, C4, P3, P4, O1, O2, F7, F8, Fz, Pz, T5, and T6) were used in the electrode arrangement called the International 10–20 System. EEG activity was measured using the binaural connection as a reference. EEG-4518 manufactured by Nihon Kohden Co., Ltd. Tokyo, Japan, was used for measurement. Eye movements were tracked using bipolar electrocardiography (EOG). The EEG signal was recorded using a sampling frequency of 200 Hz and bandpass filtered at 2.0–60 Hz. As pre-processing steps were not conducted (i.e., filtering except for bandpass, artifacts removal, or data reconstruction), because such processing may destroy the data's intrinsic dynamics, we visually selected epochs without artifacts. The electrode/skin conductance impedance was carefully controlled at each electrode to <5kΩ. Each subject's EEG signal was measured for 10–15 min in a resting state with eyes closed. A video surveillance system was used to visually inspect the subjects' alertness and to confirm that only epochs with closed eyes and a wakefulness state (not light sleep) were measured. Visual inspection of EEG and EOG records identified EEG time series segments recorded in a wakefulness state with closed eyes. Subjects were considered fully awake when predominant alpha activity appeared in the posterior region in response to the fast eye movements of the EOG channel (Wada et al., 1996). MSE analysis and MF analysis were conducted against a continuous 50-s(10000 data points) epoch.

### 2.3. Multifractal Analysis

In MF analysis, wavelet readers derived from the coefficients of the discrete wavelet transform are widely used (Jaffard et al., 2006; Wendt and Abry, 2007). The discrete wavelet coefficient of the discrete signal *X*(*t*) is given by

(1) $$\begin{array}{l} d_{X}(j,k)=\int_{R}X(t)2^{j},\psi_{0}(2^{-j}t-k)dt\;(j=1,2,...,k=1,2,...), \end{array}$$

where ψ_0_ is a compact-supported mother wavelet function. One-dimensional wavelet leaders were expressed by

(2) $$\begin{array}{l} L_{x}\left(j,k\right)=\underset{\lambda^{\prime }\subset 3\lambda_{j,k}}{\sup}|d_{X}\left(j,k\right)|. \end{array}$$

Here, $\lambda =\lambda_{j,k}=\left[k2^{j},\left(k+1\right)2^{j}\right]$ represents the time interval of scale 2^*j*^, and 3λ_*j,k*−1_ = ∪ λ_*j,k*_ ∪ λ_*j,k*+1_ represents the adjacent time (Wendt and Abry, 2007). The singular value spectrum *D*(*h*), which is the distribution of the fractal dimension represented by the Hölder exponent *h*, is represented by wavelet leaders (Jaffard et al., 2006; Wendt and Abry, 2007):

(3) $$\begin{array}{l} D\left(h\right)=\underset{q\neq 0}{\inf}\left(1+qh-\zeta_{L}\left(q\right)\right). \end{array}$$

Here, *q* indicates the moment for scaling index ζ_*L*_(*q*). The scaling index ζ_*L*_(*q*) and the structural function *S*_*L*_(*q, j*) are represented by Equations (4, 5), respectively:

(4) $$\begin{array}{l} \zeta_{L}\left(q\right)=\underset{j\to 0}{lim inf}\left(\frac{\log_{2}S_{L}\left(q,j\right)}{j},\right) \end{array}$$

(5) $$\begin{array}{l} S_{L}\left(q,j\right)=\frac{1}{n_{j}}\overset{n_{j}}{\underset{k=1}{\sum }}|L_{X}\left(j,k\right){|}^{q}. \end{array}$$

**Figure 1:**
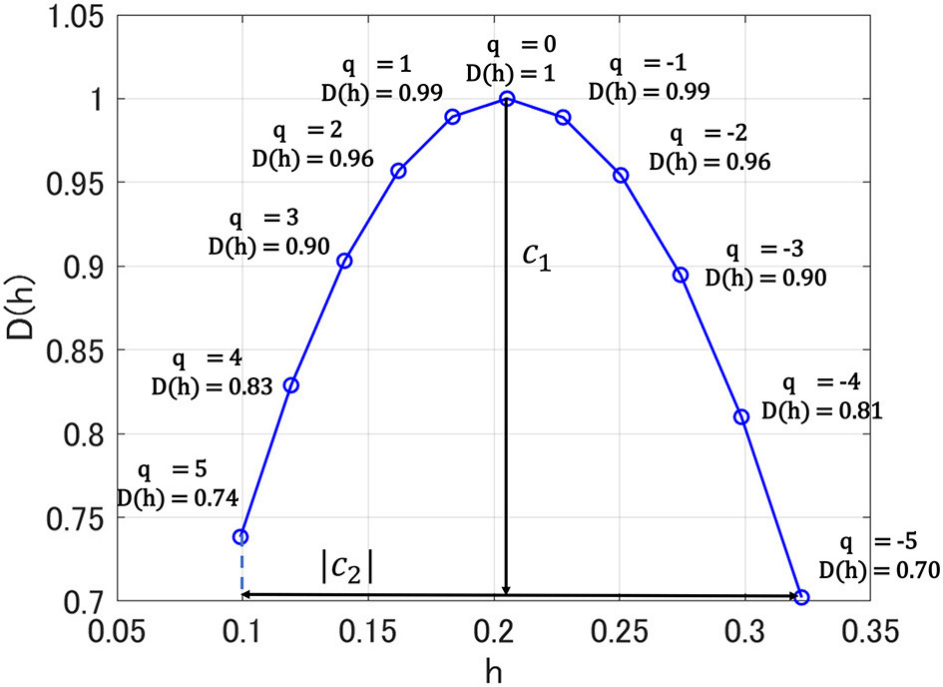
Singular value spectra *D*(*h*) in multi-fractal analysis for one healthy control (HC) subject. Here, *h* exhibits Hölder exponent. *c*_1_ shows *h*-value where *D*(*h*) = 1.0 (*q* = 0); absolute value of *c*_2_ corresponds to the range of *D*(*h*) distribution between *q* = −5 and 5.

Here, *n*_*j*_ indicates the number of samples of *X* when the scale is 2^*j*^. As Hölder exponent *h* approaches 1.0, the shape of the time series becomes more differentiable. In contrast, as Hölder exponent *h* approaches zero, the shape of the time series becomes nearly discontinuous. If the scaling index ζ_*L*_(*q*) is a linear function and *D*(*h*) converges to a particular *h*, then the signal is monofractal. On the other hand, in the scaling index, where ζ_*L*_(*q*) deviates from linearity and *D*(*h*) is distributed over a wide range of *h*, the signal is multifractal. In this study, to capture the profile of *D*(*h*), we used the primary cumulant *c*_1_ of *D*(*h*), which corresponds to a dominant component of *D*(*h*) as smoothness index estimated in the entire time-series. Moreover, we used the secondary cumulant *c*_2_, which corresponds to the magnitude of fluctuation intermittently appearing as the index for multifractality. Figure 1 shows the results of multi-fractal analysis of one HC subject. *c*_1_ shows *h*-value where *D*(*h*) = 1.0 (*q* = 0), which corresponds to the degree of differentiability in the dominant component of the entire time-series, i.e., smoothness. The absolute value of *c*_2_ corresponds to the range of *D*(*h*) distribution between *q* = −5 and 5. In the monofractal time-series, *D*(*h*) converges at a particular *h* (in the time-series with no multifractality, *D*(*h*) converges at *h*-value with *q* = 2), while in the multifractal time-series, the range of *D*(*h*) becomes wider (Ihlen, 2012; Mukli et al., 2015). Therefore, the degree of variation of *D*(*h*) corresponding to *c*_2_ reflects the multifractality. In this study, multifractal analysis was performed using the wavelet toolbox of MATLAB (https://jp.mathworks.com/products/wavelet.html).

### 2.4. Multiscale Entropy Analysis

To perform the multiscale entropy (MSE) analysis, we used the dependence of the EEG time series complexity on the temporal scale (Costa et al., 2002). The sample entropy for the time-series of random Z-scored variable {*x*_1_, *x*_2_, ..., *x*_*N*_} is defined as

(6) $$\begin{array}{l} h\left(r,m\right)=-\log\frac{C_{m+1}\left(r\right)}{C_{m}\left(r\right)}. \end{array}$$

*C*_*m*_(*r*) is the probability of $|{\text{x}}_{i}^{m}-{\text{x}}_{j}^{m}|<r\left(i\neq j,i,j=1,2,...\right)$. ${\text{x}}_{i}^{m}$ indicates an *m*-dimensional vector ${\text{x}}_{i}^{m}=\left\{x_{i},x_{i+1},...,x_{i+m-1}\right\}$. {*x*_*i*_, *x*_*i*+1_, ..., *x*_*N*_} is obtained course-grained process:

(7) $$\begin{array}{l} x_{j}=\frac{1}{\tau }\overset{j_{\tau }}{\underset{i=\left(j-1\right)\tau +1}{\sum }}y_{i}\left(1\leq j\leq \frac{N}{\tau }\right). \end{array}$$

where, {*y*_1_, *y*_2_, ..., *y*_*N*_} is observed signals. τ(τ = 1, 2, ...) is the temporal scale. In this study, we set *m* = 2 and *r* = 0.2 (Costa et al., 2002). In this study, MSE analysis was performed using the Physio Toolkit, a toolbox of MATLAB (http://physionet.incor.usp.br/physiotools/sampen/).

### 2.5. Statistical Analysis

For *c*_1_ and *c*_2_, repeated measures analysis of variance (ANOVA) with the groups (HC vs. AD) as the between-subject factor and the electrodes (16 electrodes from Fp1 to T6) as the within-subject factors was performed to test for group differences. The result of ANOVA is represented by *F*-value based on a comparison of variances within/between groups. The Greenhouse-Geisser adjustment was applied in degrees of freedom. The α bilateral level of 0.05 was used, considered a statistically significant criterion to avoid type I errors. *Post-hoc* *t*-tests were used to assess the significant main effects of group and per-electrode interactions. Benjamini–Hochberg false discovery rate (FDR) correction was applied to the *t*-score for multiple comparisons in *c*_1_ and *c*_2_ (*q* < 0.05) (16 *p*-values: 16 electrodes).

For sample entropy, repeated measures ANOVA with groups (HC vs. AD) as the between-subject factor and electrode (16 electrodes from Fp1 to T6) and temporal scale (30 temporal scales) as within-subject factors, was performed to test for group differences. The Greenhouse-Geisser adjustment and α bilateral level of 0.05 were applied. The result of ANOVA is represented by *F*-value based on a comparison of variances within/between groups. *Post-hoc* *t*-tests were used to assess the significant main effects of the group and per-electrode and per-temporal-scale interactions. The FDR correction was applied to the *t*-score for multiple comparisons (*q* < 0.05) (480 *p*-values: 16 electrode × 30 scales).

Receiver operating characteristic (ROC) curves were used to evaluate the ability to identify AD. To identify AD, a logistic regression model based on the sample entropy, *c*_1_ and *c*_2_, was used. Here, the logistic regression model outputs the identification probability of AD for each subject. Subsequently, the true positive rate/false positive rate at each threshold of identification probability from 0 to 1.0 in both groups are measured. Principal component analysis is used as a preprocess for dimensionality reduction. Logistic regression was applied to the 1st–3rd principal components of each evaluation index. The identification accuracy was evaluated by measuring the area under the ROC curve (AUC), which is an index of identification accuracy. Subsequently, according to AUC values, the classification accuracy is graded in logistic regression models based on the sample entropy, *c*_1_ and *c*_2_. Here, AUC = 1.0 corresponds to complete identification, and AUC = 0.5 corresponds to random identification.

## 3. Results

### 3.1. Multifractal Analysis

**Figure 2:**
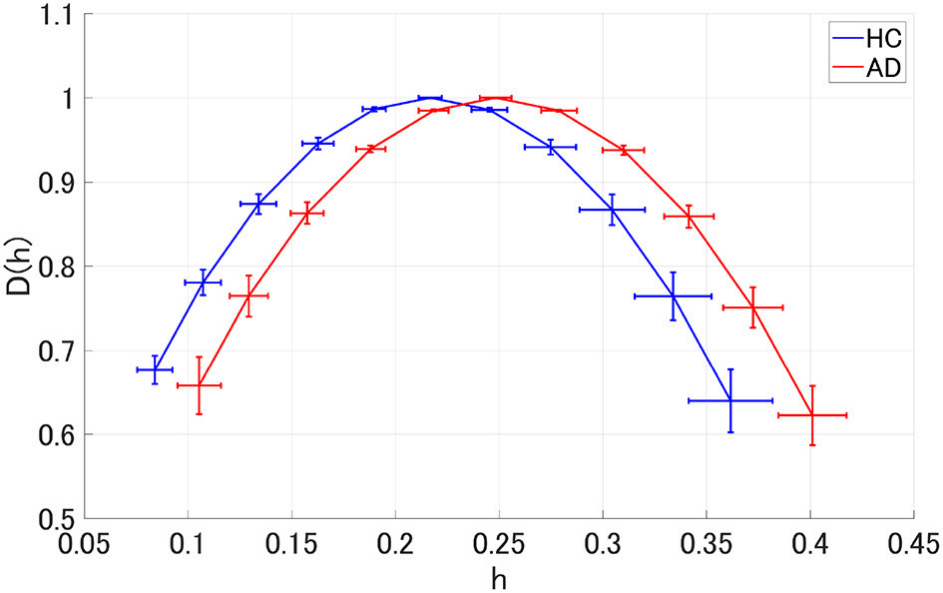
Singular value spectra of Alzheimer's disease (AD) group and HC group. The mean and standard deviation among each group of *D*(*h*) and *h*. Since the distribution is wide, it is considered that it reflects the multi-fractal property of both groups' EEG signal.

**Figure 3:**
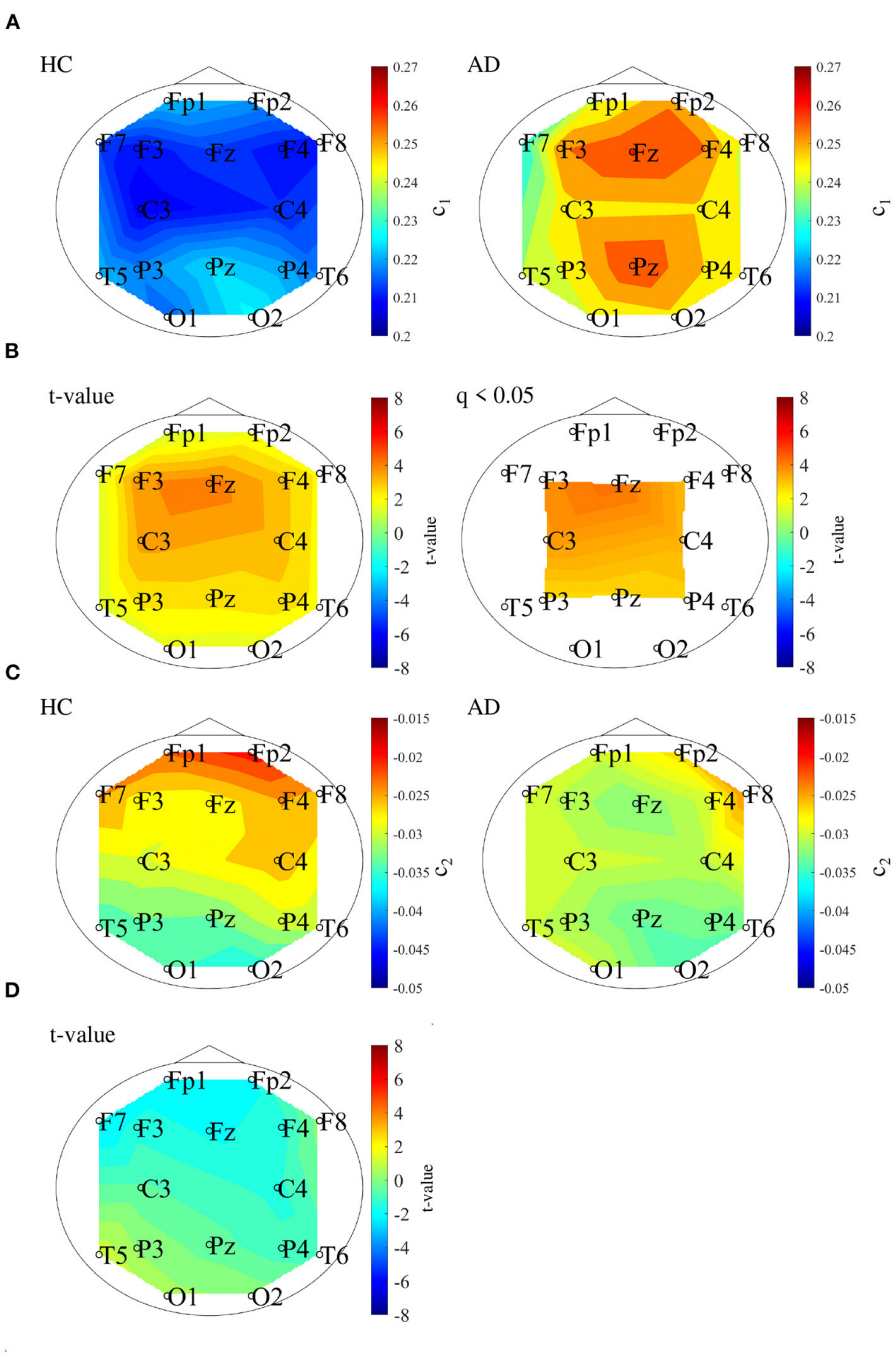
**(A)** 1st cumulant of singular value spectrum *c*_1_. Mean value of *c*_1_ in the HC (left) and AD (right) groups. **(B)** *t*-values between the AD and HC groups. The warm (cold) color represents higher (smaller) *c*_1_ values of AD than those for HC. The left and right correspond to the *t*-value and *t*-value satisfying the false discovery rate (FDR) correction criteria *q* < 0.050 corresponding to (*p* < 0.012). *c*_1_ of the AD group had significantly higher values at F3, Fz, F4, C3, C4, P3, Pz, and P4. **(C)** 2nd cumulant of singular value spectrum *c*_2_. Mean value of *c*_2_ in the HC (left) and AD (right) groups. **(D)** *t*-value between the AD and HC groups warm (cold) color represents higher (smaller) *c*_2_ values of AD than those for HC. There are no-significant high/low *t*-values satisfying FDR correction criteria *q* < 0.05 (corresponding to *p* < 0.003).

We performed MF analysis on both HC and AD groups. Figure 2 shows the mean and standard deviation for each group of *D*(*h*) and *h*. Since the distribution is wide, it reflects the multi-fractal property (Sikdar et al., 2018) of both groups' EEG signal. Table 2 shows the repeated measures ANOVA results of 1st (*c*_1_) and 2nd (*c*_2_) cumulants of singular spectrum. The significant main effect in *c*_1_ was confirmed. The mean values of *c*_1_ and *c*_2_ in the AD and HC groups and the result of the *post-hoc* *t*-test between AD and HC are shown in Figure 3. The significantly higher *c*_1_ values in the AD group (*q* < 0.050 corresponding to *p* < 0.012) was confirmed at F3, Fz, F4, C3, C4, P3, Pz, and P4.

**Table 2:** AD vs. HC repeated measure ANOVA analysis results [*F*-value (*p* value)] in multifractal (MF) analysis results, *F* and *p* value with *p* < 0.05 are represented by bold characters.

	**Group**	**Group × nodes**
c1	*F* = 9.088 (*p* = 0.005)	*F* = 1.460 (*p* = 0.204)
c2	*F* = 0.654 (*p* = 0.425)	*F* = 1.981 (*p* = 0.072)

### 3.2. Multi Scale Entropy Analysis

**Figure 4:**
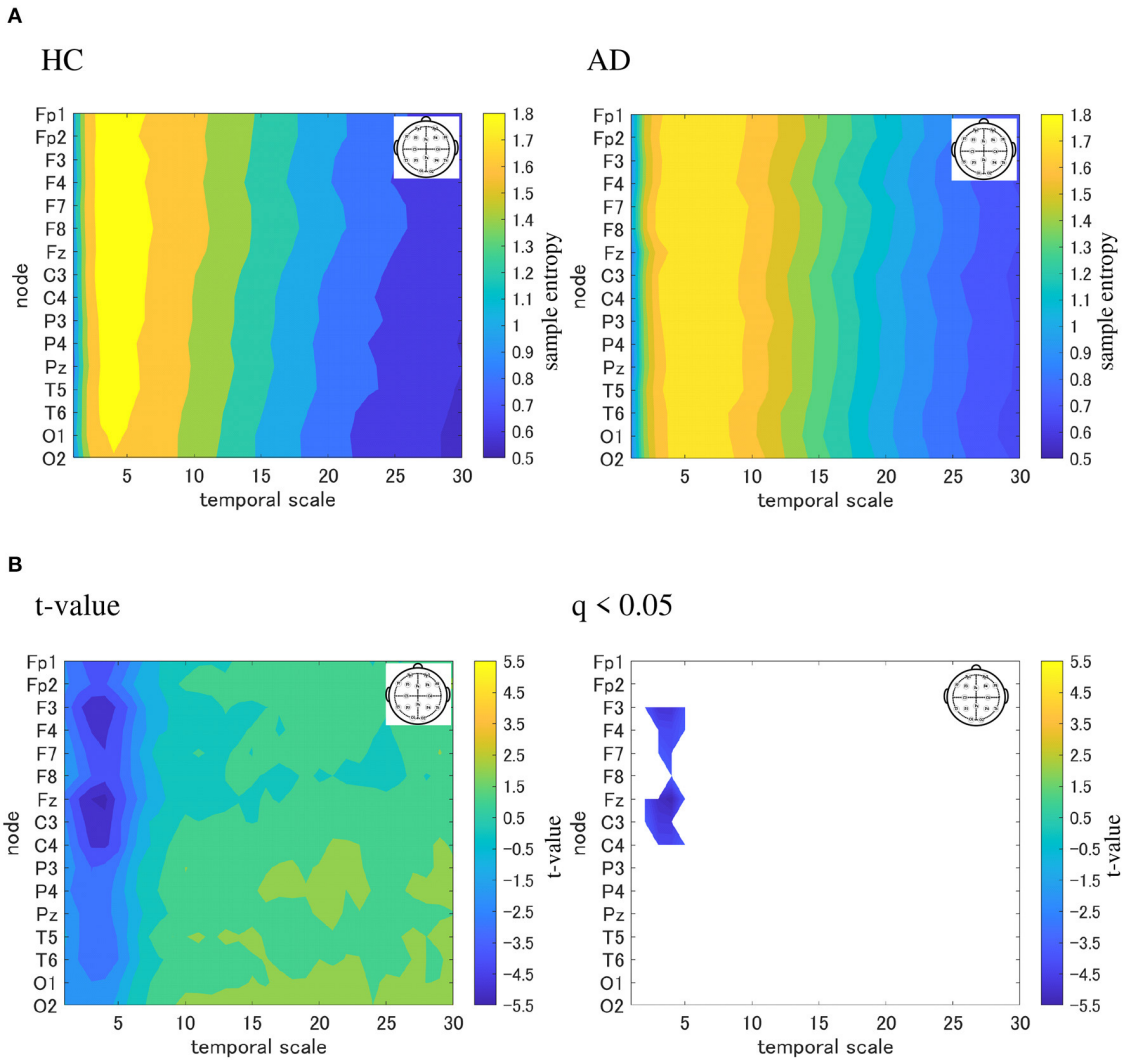
Multi-scale entropy analysis in HC and AD group. The horizontal axis represents the temporal-scale factor, τ . **(A)** Mean values of sample entropy from 1 (0.005 s) to 30 (0.15 s) scale factors in HC (left part) and AD (right part). **(B)** *t*-value between the AD and HC groups(left part). The warm (cold) color represents a higher (smaller) sample entropy value for AD than that for HC. The *t*-value satisfying the FDR correction criteria *q* < 0.050 corresponding to (*p* < 0.002). Significantly smaller sample entropy of AD low temporal scale regions 1 to 5 (0.005 to 0.025 s).

We performed an MSE analysis in the HC and AD groups. Table 3 shows the repeated measures ANOVA results of MSE analysis. Significant group × scale interactions without the main effect of sample entropy were confirmed. As *post hoc* *t*-test, the mean values of sample entropy in HC and AD groups and the *t*-value between HC and AD are shown in Figure 4. The results demonstrated a significantly lower sample entropy of AD (*q* < 0.050 corresponding to *p* < 0.002) in the temporal scale region 1 to 5 (0.005 to 0.025 s). The result of MSE analysis was reported in our previous studies (Mizuno et al., 2010; Nobukawa et al., 2020). Particularly, in the study by Mizuno et al. (2010), multiscale entropy analysis against AD EEG signals was reported, while our recent study (Nobukawa et al., 2020) showed the relationship between functional connectivity characterized by phase synchronization and multiscale entropy in AD EEG signals.

**Table 3:** AD vs. HC repeated measure ANOVA results [*F*-value (*p*-value)] in multi scale entropy (MSE) analysis results, *F* and *p* value with *p* < 0.05 are represented by bold characters.

	**Group**	**Group × node**	**Group × scale**	**Group × node × scale**
	*F* = 1.233 (*p* = 0.275)	*F* = 1.860 (*p* = 0.129)	*F* = 11.457 (*p* = 0.003)	*F* = 0.979 (*p* = 0.451)

### 3.3. ROC Curve

**Figure 5:**
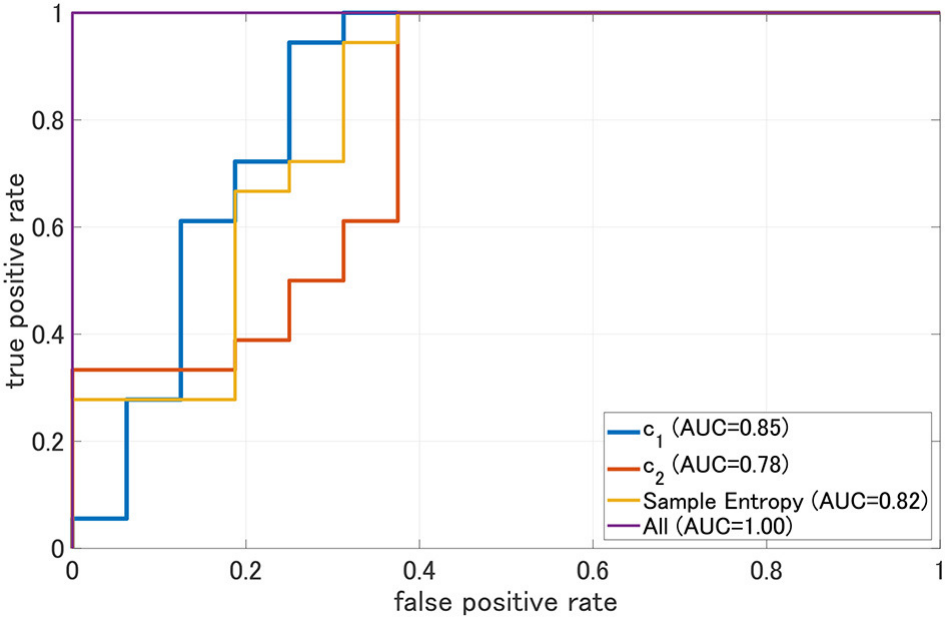
Receiver operating characteristic curve (ROC) for c1, c2, and sample entropy. The area under the ROC curve (AUC) is shown in the legend. As classifier, logistic regression is used. In this case, *c*_1_, *c*_2_, and sample entropy, each 1st-3rd principal component was used separately. In the case represented by “ALL, all 1st–3rd principal components component of *c*_1_, *c*_2_, sample entropy were used. We evaluated ROC in the case using all these values; the results show the enhancement of classification ability (AUC = 1.00).

**Figure 6:**
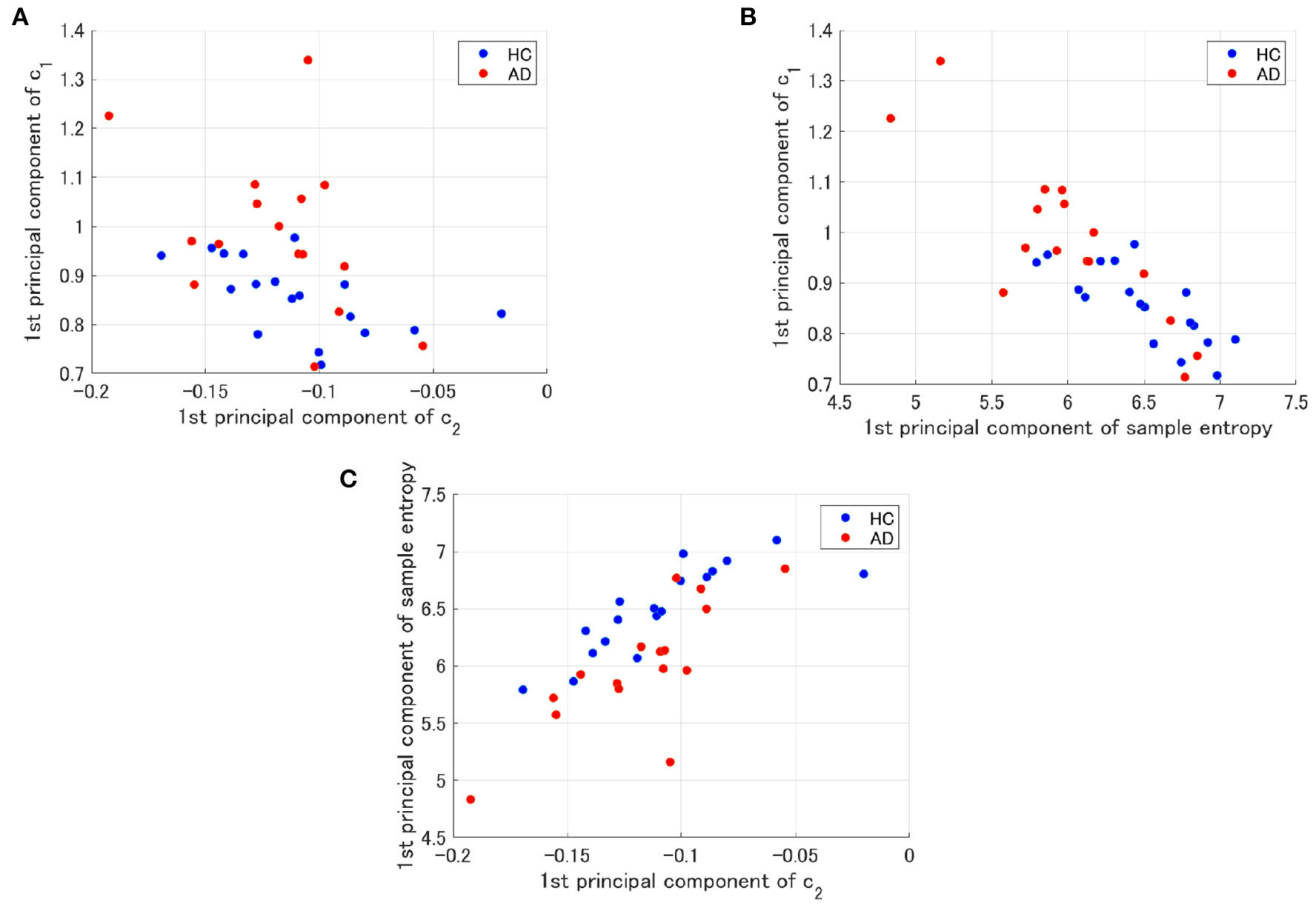
Scatter plots for 1st principal component of *c*_1_, *c*_2_ and sample entropy used to evaluate of ROC in Figure 5. **(A)** Scatter plots between *c*_1_ and *c*_2_. The result showed a relatively low negative correlation (*R* = −0.56(HC),*R* = −0.42(AD)). **(B)** Scatter plots between *c*_1_ and the sample entropy. The results showed a high negative correlation [*R* = −0.77(HC),*R* = −0.85(AD)]. **(C)** Scatter plots of the between sample entropy and *c*_2_. The result showed a positive correlation [*R* = 0.82(HC),*R* = 0.78(AD)].

**Figure 7:**
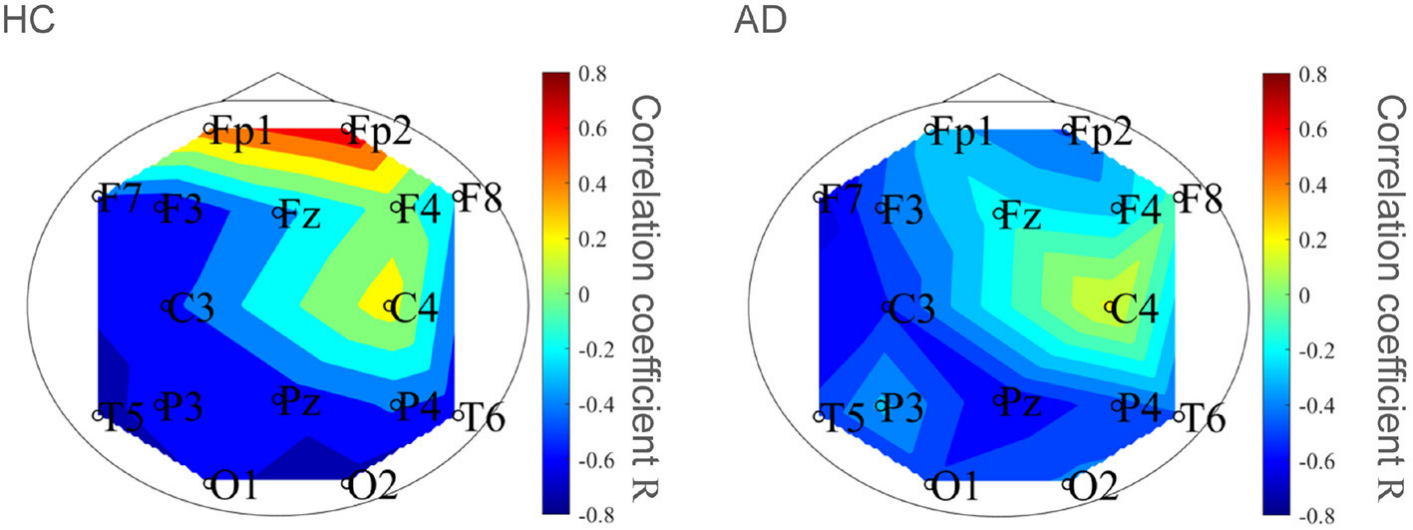
Correlation coefficient *R* between *c*_1_ and *c*_2_ in HC and AD groups. Spatial dependency of correlation coefficient *R* was confirmed, which might contribute the enhancement of classification accuracy shown in Figure 5.

To evaluate the classification ability in *c*_1_ and *c*_2_, we evaluated ROC curves. Figure 5 shows the result of ROC in the case with 1st–3rd principal components in each evaluated index. In the sample entropy case, the values are averaged in 1 to 5 temporal scale. AUC in the *c*_1_ case exhibits the highest classification ability (AUC = 0.85 in the case *c*_1_; AUC = 0.78, in the case of *c*_2_; AUC = 0.82 in the case sample entropy). Furthermore, we evaluated ROC using all these values; the results showed an enhancement of classification ability (AUC = 1.00). To investigate why the combination of *c*_1_, *c*_2_, and sample entropy enhances classification ability, we evaluated their relationship by correlation analysis. Figure 6 shows a scatterplot among the 1st component of *c*_1_, *c*_2_ and sample entropy used for ROC evaluation in Figure 5. The correlation coefficients *R* are shown in Table 4. The results show a high negative and positive correlation between *c*_1_ and sample entropy, a positive correlation between *c*_2_ and sample entropy, and a relatively low negative correlation between *c*_1_ and *c*_2_. This relatively low correlation between *c*_1_ and *c*_2_ suggests that *c*_2_ includes complementary information regarding multifractality in the classification. Moreover, to investigate the correlation between *c*_1_ and *c*_2_, not the principal components, the correlation coefficient *R* between *c*_1_ and *c*_2_ in HC and AD groups is demonstrated in Figure 7. The results show the spatial dependency of correlation coefficient *R*, which might contribute to the enhancement of classification accuracy shown in Figure 7.

**Table 4:** Correlation coefficient values (*R*) for each combination of *c*_1_, *c*_2_, and sample entropy in HC and AD.

***c*_1_ vs. *c*_2_**	***c*_1_** **vs. Sample entropy**	**Sample entropy vs. *c*_2_**	
Correlation coefficient(HC)	*R* = −0.56	*R* = −0.77	*R* = 0.82
Correlation coefficient(AD)	*R* = −0.42	*R* = −0.85	*R* = 0.78

**Figure 8:**
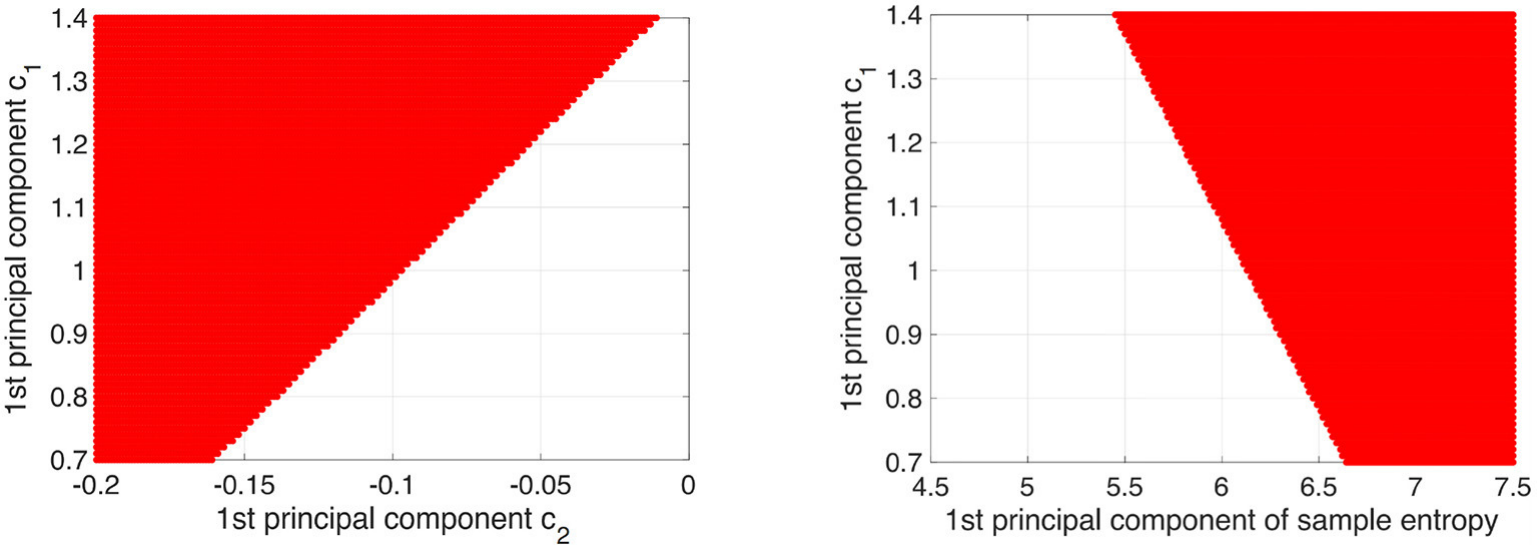
Decision region (given by red region) for AD with decision probability more than 0.9 by logistic regression model was shown on the plane between 1st principal component of *c*_1_ and 1st principal component of *c*_2_ (left part) and plane between 1st principal component of *c*_1_ and 1st principal component of sample entropy (right part). Here, the other components except axis of planes were set to average among subjects in both HC and AD groups. The dependency on all of them in the decision region was confirmed.

To demonstrate that the decision region for AD is determined by *c*_1_, *c*_2_, and sample entropy, decision regions for AD with decision probability >0.9 by logistic regression model were depicted on the plane between the 1st principal component of *c*_1_ and the 1st principal component of *c*_2_ and the plane between the 1st principal component of *c*_1_ and the 1st principal component of the sample entropy (see Figure 8). Here, all other components except the plane axis were set to average among subjects in both the HC and AD groups. As a result, we confirmed that the decision region exhibits dependent on all of them.

## 4. Discussions

This study evaluated AD identification accuracy by focusing on the complementary relationship between two complexity analyses. The MF and MSE of EEG signals in HC and AD were evaluated, and classification accuracies quantified by the AUC of logistic regression models were compared. The results of *c*_1_ as the index for the smoothness of the EEG time series by MF analysis showed that *c*_1_ of AD significantly increased. However, the results of *c*_2_, as the index for the EEG time series' multifractal nature by MF analysis, show that no significant AD alteration was observed. MSE analysis showed a significant region-specific reduction of small-temporal-scale sample entropy of AD (corresponding to the complexity of faster temporal EEG behaviors). In the comparison of classification accuracy between *c*_1_, *c*_2_, and small-temporal-scale sample entropy, *c*_1_ exhibits the highest classification accuracy. Moreover, the classification accuracy with *c*_1_ was enhanced by considering the complementary relationship of *c*_2_ and sample entropy.

We must discuss the reason why *c*_1_ as the degree of smoothness increase in AD. In the alteration of EEG/MEG signals in AD, a reduction in temporal complexity has been widely observed (Woyshville and Calabrese, 1994; Besthorn et al., 1995; Jelles et al., 1999; Jeong, 2004; Wickramasinghe and Geisler, 2008; Smits et al., 2016; Kulkarni, 2018; Smailovic et al., 2019). Correspondingly, our results of sample entropy at a small temporal scale also exhibited a reduction in complexity. Considering the negative correlation between *c*_1_ and the small-temporal-scale sample entropy (see Table 4), the increase in *c*_1_, that is, the enhancement of EEG signal smoothness in AD, was caused by the decrease in small-temporal-scale complexity. Therefore, the enhancement of *c*_1_ reflects the loss of temporal complexity of neural activity in AD. This finding agrees with previous studies on MF analysis in AD (Jaffard et al., 2006; Wendt and Abry, 2007).

Furthermore, we must consider why small temporal scale complexity decreases in AD. Dysfunction of the gamma-aminobutyric acid (GABA) signaling system caused by deposition of amyloid β and tau protein have been reported. These changes lead to the reduced oscillation of the gamma band activity produced by GABA signaling (Nava-Mesa et al., 2014; Govindpani et al., 2017; Calvo-Flores Guzmán et al., 2018). Consequently, dysfunction of the mutual interaction of gamma band activity can reduce the complexity more on the faster than on the slower temporal scales (Ahmadlou et al., 2011; Nobukawa et al., 2019).

Next, it is necessary to consider why the classification accuracy was highest when *c*_1_, *c*_2_, and sample entropy were used. According to Cukic *et al*., sample entropy and fractal dimension by mono-fractal analysis show a complementary relationship among temporal scales (Cukic et al., 2018), and this relationship can enhance the ability to detect an alteration of complexity. Our results (see Figure 8) also showed that a decision region for AD with decision probability >0.9 by logistic regression model exhibits a dependency on *c*_1_, *c*_2_, and sample entropy. Therefore, the combination of *c*_1_ corresponding to the fractal dimension and sample entropy might enhance the accuracy of AD detection. In addition to these findings, a recent MF analysis in AD showed that the multifractal degree reflects disease-specific alterations of complexity (Zorick et al., 2020). Although the classification ability in case of separate use of multifractals measured by *c*_2_ is relatively low, the combination with *c*_2_ may contribute to the improvement of classification accuracy.

To investigate whether the high heterogeneity of severity in patients with AD affects classification, we investigated distributions of *c*_1_, *c*_2_, and mean sample entropy in a scale from 1 to 5 according to severity as classified by FAST (3 (mild dementia), 4 (moderate dementia), and 5 (severe dementia), through repeated measures ANOVA with severity as a between-subject factor and electrode as a within-subjects factor. The results showed that severity did not have any significant main effect or interaction in *c*_1_ (*F* = 0.412, *p* = 0.671), *c*_2_ (*F* = 0.706, *p* = 0.512), and sample entropy (*F* = 0.532, *p* = 0.6); while a significant interaction between severity and electrodes in *c*_1_ (*F* = 2.103, *p* = 0.036) appeared. Therefore, although in larger AD groups a severity-dependent effect may appear, the bias of high heterogeneity of severity is limited. Additionally, in patients with mild dementia, no differences in the distribution of *c*_1_, *c*_2_, and sample entropy compared to more severe patients appeared in the repeated measures ANOVA. Therefore, the classification accuracy may not change in case of a classification between HC and patients with mild dementia, which corresponds to the condition assumed for early diagnosis.

Finally, we must consider the limitations of this study. First, EEG signals do not always reflect the neural activity directly under the electrode. In this study, 16 electrodes were used to measure EEG, but the spatial resolution was too low to identify AD's complex functional connection structure. However, it is possible to use MEGs with a high spatial resolution and cortical positioning to solve this problem. Second, pre-processing for EEG signals was not adopted except for a band-pass filter. However, a recent study by Racz et al. (2018) indicated that appropriate pre-processing is needed for complexity analysis. Artifacts and noise are to be avoided, especially at the stage of clinical application. Therefore, this pre-processing for complexity analysis must be developed and adopted in future studies. Third, we consider that for our EEG data set, the multifractal analysis method proposed by Jaffard et al. (2006) and Wendt and Abry (2007) is sufficient, because a corrupted/inversed *D*(*h*) distribution did not arise (see Figure 2). Additionally, this study was conducted on the assumption of multifractality in EEG signals (Takahashi, 2013; Yang and Tsai, 2013; Sikdar et al., 2018). However, several studies highlighted the issues of incorrect estimation of multifractal indexes in time-series without multifractality (Grech and Pamula, 2012; Mukli et al., 2015). Therefore, multifractal analysis methods with higher robustness (Mukli et al., 2015) are desired at the stage of clinical application, since proper validation of EEG multifractality (Mukli et al., 2015; Racz et al., 2018) is an important issue. Fourth, the AD group had high heterogeneity of severity, and the sample size of the AD group was small, which could have influenced the classification accuracy. Therefore, the classification ability of our proposed method must be evaluated in larger AD groups. Additionally, a Bayesian statistic approach is more suitable for small size and high sample heterogeneity than that based on frequentist inference.

## 5. Conclusion

In this study, both MSE and MF analysis showed a reduction in EEG complexity in AD patients. Classification accuracy is better by combining MSE analysis and MF analysis than when applying each one individually. Despite its limitations, this study shows that MSE and MF analysis play complementary roles in detecting the alteration of neural activity in AD. The use of both MSE and MF analysis may facilitate the development of AD diagnostic tools.

## Data Availability Statement

The datasets presented in this article are not readily available because the informed consent did not include the declaration regarding publicity of clinical data. Requests to access the datasets should be directed to Sou Nobukawa, nobukawa@cs.it-chiba.ac.jp.

## Ethics Statement

The studies involving human participants were reviewed and approved by Ethics Committee of Kanazawa University. The patients/participants provided their written informed consent to participate in this study.

## Author Contributions

MA, SN, MK, and TT conceived the methods. MA and SN analyzed the results, wrote the main manuscript text, and prepared all the figures. MK conducted the experiments. All authors reviewed the manuscript.

## Conflict of Interest

The authors declare that the research was conducted in the absence of any commercial or financial relationships that could be construed as a potential conflict of interest.

## References

[B1] Abásolo D. Escudero J. Hornero R. Gómez C. Espino P. (2008). Approximate entropy and auto mutual information analysis of the electroencephalogram in Alzheimer's disease patients. Med. Biol. Eng. Comput. 46, 1019–1028. 10.1007/s11517-008-0392-1 18784948

[B2] Ahmadlou M. Adeli H. Adeli A. (2011). Fractality and a wavelet-chaos-methodology for EEG-based diagnosis of Alzheimer disease. Alzheimer Dis. Assoc. Disord. 25, 85–92. 10.1097/WAD.0b013e3181ed1160 20811268

[B3] Al-Nuaimi A. H. Jammeh E. Sun L. Ifeachor E. (2017). Higuchi fractal dimension of the electroencephalogram as a biomarker for early detection of Alzheimer's disease, in 2017 39th Annual International Conference of the IEEE Engineering in Medicine and Biology Society (EMBC) (Jeju: IEEE), 2320–2324. 10.1109/EMBC.2017.8037320 29060362

[B4] Besthorn C. Sattel H. Geiger-Kabisch C. Zerfass R. Förstl H. (1995). Parameters of eeg dimensional complexity in Alzheimer's disease. Electroencephalogr. Clin. Neurophysiol. 95, 84–89. 10.1016/0013-4694(95)00050-9 7649009

[B5] Brookmeyer R. Johnson E. Ziegler-Graham K. Arrighi H. M. (2007). Forecasting the global burden of Alzheimer's disease. Alzheimer's Dement. 3, 186–191. 10.1016/j.jalz.2007.04.381 19595937

[B6] Calvo-Flores Guzmán B. Vinnakota C. Govindpani K. Waldvogel H. J. Faull R. L. Kwakowsky A. (2018). The gabaergic system as a therapeutic target for Alzheimer's disease. J. Neurochem. 146, 649–669. 10.1111/jnc.14345 29645219

[B7] Costa M. Goldberger A. L. Peng C.-K. (2002). Multiscale entropy analysis of complex physiologic time series. Phys. Rev. Lett. 89, 068102. 10.1103/PhysRevLett.89.068102 12190613

[B8] Cukic M. B. Platisa M. M. Kalauzi A. Oommen J. Ljubisavljevic M. R. (2018). The comparison of Higuchi fractal dimension and Sample Entropy analysis of sEMG: effects of muscle contraction intensity and TMS. arXiv [Preprint] arXiv:1803.10753.

[B9] Dickerson B. C. Sperling R. A. (2008). Functional abnormalities of the medial temporal lobe memory system in mild cognitive impairment and Alzheimer's disease: insights from functional MRI studies. Neuropsychologia 46, 1624–1635. 10.1016/j.neuropsychologia.2007.11.030 18206188 PMC2760288

[B10] Ewers M. Sperling R. A. Klunk W. E. Weiner M. W. Hampel H. (2011). Neuroimaging markers for the prediction and early diagnosis of Alzheimer's disease dementia. Trends Neurosci. 34, 430–442. 10.1016/j.tins.2011.05.005 21696834 PMC3275347

[B11] Gómez C. Vaquerizo-Villar F. Poza J. Ruiz S. J. Tola-Arribas M. A. Cano M.. (2017). Bispectral analysis of spontaneous EEG activity from patients with moderate dementia due to Alzheimer's disease, in 2017 39th Annual International Conference of the IEEE Engineering in Medicine and Biology Society (EMBC) (Jeju: IEEE), 422–425. 10.1109/EMBC.2017.8036852 29059900

[B12] Govindpani K. Calvo-Flores Guzmán B. Vinnakota C. Waldvogel H. J. Faull R. L. Kwakowsky A. (2017). Towards a better understanding of gabaergic remodeling in Alzheimer's disease. Int. J. Mol. Sci. 18, 1813. 10.3390/ijms18081813 28825683 PMC5578199

[B13] Grech D. Pamula G. (2012). Multifractal background noise of monofractal signals. Acta Phys. Pol. A 121, 34–39. 10.12693/APhysPolA.121.B-34

[B14] Greicius M. D. Srivastava G. Reiss A. L. Menon V. (2004). Default-mode network activity distinguishes Alzheimer's disease from healthy aging: evidence from functional mri. Proc. Natl. Acad. Sci. U.S.A. 101, 4637–4642. 10.1073/pnas.0308627101 15070770 PMC384799

[B15] Ieracitano C. Mammone N. Hussain A. Morabito F. C. (2020). A novel multi-modal machine learning based approach for automatic classification of EEG recordings in dementia. Neural Netw. 123, 176–190. 10.1016/j.neunet.2019.12.006 31884180

[B16] Ihlen E. A. F. E. (2012). Introduction to multifractal detrended fluctuation analysis in matlab. Front. Physiol. 3:141. 10.3389/fphys.2012.00141 22675302 PMC3366552

[B17] Jaffard S. Lashermes B. Abry P. (2006). Wavelet leaders in multifractal analysis, in Wavelet Analysis and Applications (Basel: Springer), 201–246.

[B18] Jelles B. Van Birgelen J. Slaets J. Hekster R. Jonkman E. Stam C. (1999). Decrease of non-linear structure in the EEG of Alzheimer patients compared to healthy controls. Clin. Neurophysiol. 110, 1159–1167. 10.1016/S1388-2457(99)00013-9 10423182

[B19] Jeong J. (2004). Eeg dynamics in patients with Alzheimer's disease. Clin. Neurophysiol. 115, 1490–1505. 10.1016/j.clinph.2004.01.001 15203050

[B20] Kantz H. Schreiber T. (2004). Nonlinear Time Series Analysis Vol. 7, Cambridge: Cambridge University Press. 10.1017/CBO9780511755798

[B21] Klimesch W. Sauseng P. Hanslmayr S. Gruber W. Freunberger R. (2007). Event-related phase reorganization may explain evoked neural dynamics. Neurosci. Biobehav. Rev. 31, 1003–1016. 10.1016/j.neubiorev.2007.03.005 17532471

[B22] Kulkarni N. (2018). Use of complexity based features in diagnosis of mild Alzheimer disease using EEG signals. Int. J. Inf. Technol. 10, 59–64. 10.1007/s41870-017-0057-0

[B23] Kulkarni N. Bairagi V. (2018). EEG-Based Diagnosis of Alzheimer Disease: A Review and Novel Approaches for Feature Extraction and Classification Techniques. London; San Diego, CA; Cambridge, MA; Oxford: Academic Press.

[B24] Liu S. Liu S. Cai W. Pujol S. Kikinis R. Feng D. (2014). Early diagnosis of Alzheimer's disease with deep learning, in 2014 IEEE 11th International Symposium on Biomedical Imaging (ISBI) (IEEE), 1015–1018. 10.1109/ISBI.2014.6868045

[B25] McKhann G. M. Knopman D. S. Chertkow H. Hyman B. T. Jack C. R. Jr Kawas C. H.. (2011). The diagnosis of dementia due to Alzheimer's disease: recommendations from the national institute on aging-Alzheimer's association workgroups on diagnostic guidelines for Alzheimer's disease. Alzheimers Dement. 7, 263–269. 10.1016/j.jalz.2011.03.005 21514250 PMC3312024

[B26] Mizuno T. Takahashi T. Cho R. Y. Kikuchi M. Murata T. Takahashi K.a (2010). Assessment of eeg dynamical complexity in Alzheimer's disease using multiscale entropy. Clin. Neurophysiol. 121, 1438–1446. 10.1016/j.clinph.2010.03.025 20400371 PMC2914820

[B27] Mukli P. Nagy Z. Eke A. (2015). Multifractal formalism by enforcing the universal behavior of scaling functions. Phys. A Stat. Mech. Appl. 417, 150–167. 10.1016/j.physa.2014.09.002

[B28] Nava-Mesa M. O. Jiménez-Díaz L. Yajeya J. Navarro-Lopez J. D. (2014). Gabaergic neurotransmission and new strategies of neuromodulation to compensate synaptic dysfunction in early stages of Alzheimer's disease. Front. Cell. Neurosci. 8:167. 10.3389/fncel.2014.00167 24987334 PMC4070063

[B29] Ni H. Zhou L. Ning X. Wang L. (ADNI) A. D. N. I. (2016). Exploring multifractal-based features for mild Alzheimer's disease classification. Magn. Reson. Med. 76, 259–269. 10.1002/mrm.25853 26193379

[B30] Nobukawa S. Yamanishi T. Kasakawa S. Nishimura H. Kikuchi M. Takahashi T. (2020). Classification methods based on complexity and synchronization of electroencephalography signals in Alzheimer's disease. Front. Psychiatry 11:255. 10.3389/fpsyt.2020.00255 32317994 PMC7154080

[B31] Nobukawa S. Yamanishi T. Nishimura H. Wada Y. Kikuchi M. Takahashi T. (2019). Atypical temporal-scale-specific fractal changes in Alzheimer's disease EEG and their relevance to cognitive decline. Cogn. Neurodyn. 13, 1–11. 10.1007/s11571-018-9509-x 30728867 PMC6339858

[B32] Racz F. S. Stylianou O. Mukli P. Eke A. (2018). Multifractal dynamic functional connectivity in the resting-state brain. Front. Physiol. 9:1704. 10.3389/fphys.2018.01704 30555345 PMC6284038

[B33] Sikdar D. Roy R. Mahadevappa M. (2018). Epilepsy and seizure characterisation by multifractal analysis of EEG subbands. Biomed. Signal Proc. Control 41, 264–270. 10.1016/j.bspc.2017.12.006

[B34] Sims R. Van Der Lee S. J. Naj A. C. Bellenguez C. Badarinarayan N. Jakobsdottir J.. (2017). Rare coding variants in PLCG2, ABI3, and TREM2 implicate microglial-mediated innate immunity in Alzheimer's disease. Nat. Genet. 49, 1373–1384. 10.1038/ng.3916 28714976 PMC5669039

[B35] Smailovic U. Koenig T. Laukka E. J. Kalpouzos G. Andersson T. Winblad B.. (2019). Eeg time signature in Alzheimer´ s disease: functional brain networks falling apart. Neuroimage Clin. 24:102046. 10.1016/j.nicl.2019.102046 31795039 PMC6909352

[B36] Smits F. M. Porcaro C. Cottone C. Cancelli A. Rossini P. M. Tecchio F. (2016). Electroencephalographic fractal dimension in healthy ageing and Alzheimer's disease. PLoS ONE 11:e0149587. 10.1371/journal.pone.0149587 26872349 PMC4752290

[B37] Sperling R. A. Aisen P. S. Beckett L. A. Bennett D. A. Craft S. Fagan A. M.. (2011). Toward defining the preclinical stages of Alzheimer's disease: Recommendations from the national institute on aging-Alzheimer's association workgroups on diagnostic guidelines for Alzheimer's disease. Alzheimers Dement. 7, 280–292. 10.1016/j.jalz.2011.03.003 21514248 PMC3220946

[B38] Stam C. J. (2005). Nonlinear dynamical analysis of EEG and MEG: review of an emerging field. Clin. Neurophysiol. 116, 2266–2301. 10.1016/j.clinph.2005.06.011 16115797

[B39] Takahashi T. (2013). Complexity of spontaneous brain activity in mental disorders. Prog. Neuropsychopharmacol. Biol. Psychiatry 45, 258–266. 10.1016/j.pnpbp.2012.05.001 22579532

[B40] Vecchio F. Babiloni C. Lizio R. Fallani F. D. V. Blinowska K. Verrienti G.. (2013). Resting state cortical EEG rhythms in Alzheimer's disease: toward eeg markers for clinical applications: a review. Suppl. Clin. Neurophysiol. 62, 223–236. 10.1016/B978-0-7020-5307-8.00015-6 24053043

[B41] Wada Y. Nanbu Y. Koshino Y. Shimada Y. Hashimoto T. (1996). Inter-and intrahemispheric EEG coherence during light drowsiness. Clin. EEG Neurosci. 27, 84–88. 10.1177/155005949602700207 8681467

[B42] Wang B. Niu Y. Miao L. Cao R. Yan P. Guo H.. (2017). Decreased complexity in Alzheimer's disease: resting-state fMRI evidence of brain entropy mapping. Front. Aging Neurosci. 9:378. 10.3389/fnagi.2017.00378 29209199 PMC5701971

[B43] Wang R. Wang J. Li S. Yu H. Deng B. Wei X. (2015). Multiple feature extraction and classification of electroencephalograph signal for Alzheimers' with spectrum and bispectrum. Chaos 25, 013110. 10.1063/1.4906038 25637921

[B44] Wendt H. Abry P. (2007). Multifractality tests using bootstrapped wavelet leaders. IEEE Trans. Signal Proc. 55, 4811–4820. 10.1109/TSP.2007.896269

[B45] Wickramasinghe N. Geisler E. (2008). Encyclopedia of Healthcare Information Systems. London; Pennsylvania, PA: IGI Global. 10.4018/978-1-59904-889-5

[B46] Woyshville M. J. Calabrese J. R. (1994). Quantification of occipital eeg changes in Alzheimer's disease utilizing a new metric: the fractal dimension. Biol. Psychiatry 35, 381–387. 10.1016/0006-3223(94)90004-3 8018784

[B47] Yamaguchi-Kabata Y. Morihara T. Ohara T. Ninomiya T. Takahashi A. Akatsu H.. (2018). Integrated analysis of human genetic association study and mouse transcriptome suggests lbh and shf genes as novel susceptible genes for amyloid-β accumulation in Alzheimer's disease. Hum. Genet. 137, 521–533. 10.1007/s00439-018-1906-z 30006735 PMC6061045

[B48] Yang A. C. Tsai S.-J. (2013). Is mental illness complex? from behavior to brain. Progr. Neuropsychopharmacol. Biol. Psychiatry 45, 253–257. 10.1016/j.pnpbp.2012.09.015 23089053

[B49] Zorick T. Landers J. Leuchter A. Mandelkern M. A. (2020). Eeg multifractal analysis correlates with cognitive testing scores and clinical staging in mild cognitive impairment. J. Clin. Neurosci. 76, 195–200. 10.1016/j.jocn.2020.04.003 32307299

